# Granulosa cell-derived miR-379-5p regulates macrophage polarization in polycystic ovarian syndrome

**DOI:** 10.3389/fimmu.2023.1104550

**Published:** 2023-03-24

**Authors:** Reza Salehi, Meshach Asare-Werehene, Brandon A. Wyse, Atefeh Abedini, Bo Pan, Alex Gutsol, Sahar Jahangiri, Peter Szaraz, Kevin D. Burns, Barbara Vanderhyden, Julang Li, Dylan Burger, Clifford L. Librach, Benjamin K. Tsang

**Affiliations:** ^1^ Chronic Disease Program, Ottawa Hospital Research Institute, Ottawa, ON, Canada; ^2^ Department of Obstetrics and Gynecology, University of Ottawa, Ottawa, ON, Canada; ^3^ Department of Cellular and Molecular Medicine and Center for Infection, Immunity and Inflammation, University of Ottawa, Ottawa, ON, Canada; ^4^ CReATe Fertility Centre, Toronto, ON, Canada; ^5^ Cancer Therapeutics Program, Ottawa Hospital Research Institute, Ottawa, ON, Canada; ^6^ Department of Animal BioScience, University of Guelph, Guelph, ON, Canada; ^7^ Division of Nephrology, Department of Medicine, Kidney Research Centre, University of Ottawa, Ottawa, ON, Canada; ^8^ Department of Obstetrics & Gynaecology, University of Toronto, Toronto, ON, Canada; ^9^ Department of Physiology, University of Toronto, Toronto, ON, Canada; ^10^ Institute of Medical Sciences, University of Toronto, Toronto, ON, Canada

**Keywords:** miR-379-5p, PCOS (polycystic ovarian syndrome), macrophage, PDK1, granulosa cells, extracellular vesicle, exosomes

## Abstract

Polycystic ovarian syndrome (PCOS) is associated with hyperandrogenemia and ovarian antral follicle growth arrest. We have previously demonstrated that androgen-induced exosomal release of miR-379-5p (miR379) from preantral follicle granulosa cells increases the proliferation of target cells *via* phosphoinositide-dependent kinase 1 (PDK1) upregulation. Androgen also increases inflammatory M1 macrophage abundance, but reduces anti-inflammatory M2 polarization in rat antral and preovulatory follicles. However, the role of small extracellular vesicles (sEVs; also known as exosomes) secretion in determining the cellular content and function of miRNAs in exosome-receiving cells is largely unknown. Our objectives were to determine: 1) the regulatory role of granulosa cells (GC)-derived exosomal miR379 on macrophage polarization and ovarian inflammation; 2) whether miR379-induced M1 polarization regulates GC proliferation; and 3) if this regulated process is follicular stage-specific. Compared with non-PCOS subjects, PCOS subjects had a higher M1/M2 ratio, supporting the concept that PCOS is an inflammatory condition. Ovarian overexpression of miR379 increased the number of M1 macrophages and the M1/M2 ratio in preantral follicles specifically. Transfection of macrophages with a miR379 mimic reduced the cellular content of PDK1 and induced M0→M1 polarization; whereas its inhibitor polarized M0→M2. Conditioned media from macrophages transfected with miR379 mimic and follicular fluid from PCOS subjects had higher galectin-3 content, a pro-inflammatory cytokine which specifically suppresses human antral follicle GC proliferation. These results indicate that miR379 inhibits M2 macrophage polarization, a condition which suppresses GC proliferation in a follicle stage-dependent manner, as exhibited in PCOS.

## Introduction

Polycystic ovarian syndrome (PCOS) is a multi-factorial heterogeneous syndrome with complex pathologies. PCOS is associated with androgen excess, increased ovarian preantral follicular growth, antral follicle growth arrest and chronic anovulation (1). In PCOS subjects, the peripheral level of chronic inflammatory markers (e.g. galectin-3) is positively correlated with hyperandrogenemia (2–5). We have shown that DHT increases inflammatory M1 macrophages and the M1/M2 ratio, but reduces anti-inflammatory M2 macrophages in rat antral and preovulatory follicles (6); supporting the concept that PCOS is an inflammatory response. However, it is not known how hyperandrogenism regulates macrophage polarization in the ovaries, and if microRNAs (miRNAs) are involved in this response.

miRNAs are small non-coding RNAs which play important roles in the regulation of macrophage polarization by post-transcriptionally down-regulating target gene expression (7–9). Exosomes (also known as small extracellular vesicles) are nano-sized vesicles which measure between 30 – 200 nm, and are involved in cell-cell communication (10–12) by selectively packaging and transferring bioactive materials (e.g. miRNAs (13, 14)). We have demonstrated that 89 miRNAs, including miR-379-5p (miR379), are differentially expressed in an androgenized rat PCOS model (15). miR379 is a member of the cluster delta-like homolog 1 gene and the type III iodothyronine deiodinase gene (DLK1-DIO3) (16). Our recent findings using an androgenized rat PCOS model exhibited lower granulosa cell (GC) miR379 but higher phosphoinositide-dependent kinase-1 (PDK1; a miR379 target) content and increased proliferation (17). Androgen reduces GC miR379 content by increasing its exosome release in preantral follicles, but not in antral follicles *in vitro*. These findings suggest that increased exosomal miR379 release in GCs is a proliferative response to androgenic stimulation, specific to the preantral stage of follicle development, and that dysregulation of this response at the antral stage is associated with follicular growth arrest, as observed in human PCOS (17).

The PI3K/AKT pathway is involved in M2 macrophage polarization and inhibition of phosphatase and tensin homolog (PTEN; an inhibitor of the AKT pathway) increases the levels of Arginase 1 and M2 polarization (18, 19). Moreover, inhibition of AKT phosphorylation through knockdown of PDK1 increases M1 polarization and the susceptibility of mice to endotoxin shock (20). However, further studies are required to determine if and how the uptake GC-derived exosomal miR379 by macrophages would alter their polarization.

In this current study, our specific objectives were to examine whether: 1) miR379 regulates macrophage polarization; 2) suppressed M2-macrophage polarization inhibits GC proliferation and steroidogenesis; and 3) the above responses are follicular stage-dependent. Our overall hypothesis was that the uptake of GC-derived exosomal miR379 induces macrophage polarization to inflammatory (M1) and increases inflammatory cytokine secretion, a response which inhibits GC proliferation in the antral, but not preantral follicles.

## Materials and methods

### Reagents and antibodies

Cell culture media (M199), DMEM/F12, fetal bovine serum (FBS), penicillin and streptomycin, L-glutamine, sodium pyruvate, and trypsin were purchased from Invitrogen (Burlington, Canada). Diethylstilbestrol (DES), equine chorionic gonadotropin (eCG), HEPES and bovine serum albumin (BSA) were from Sigma (St. Louis, MO). 5α-dihydrotestosterone (DHT) was obtained from Steraloids (Newport, RI). Anti-rabbit and -mouse IgG conjugated with horseradish peroxidase and reagents for SDS-PAGE were purchased from Bio-Rad Laboratories (Mississauga, Ontario, Canada). Enhanced chemiluminescent reagent was from Thermo Fisher Scientific (Rockford, IL).

### Animals, DHT implant, bone marrow derived macrophages, macrophage polarization, GCs and miRNA transfection

All animal procedures were carried out in accordance with the Guidelines for the Care and Use of Laboratory Animals, Canadian Council on Animal Care, and were approved by the University of Ottawa Animal Care Committee. Female Sprague Dawley rats (Charles River, Montreal, Canada) were maintained on 12 h cycle (light and dark) and given food and water ad libitum. Immature female rats at 21 day of age were implanted subcutaneously with silicone capsules without (control, sham control) or with DHT (DHT, Steraloids Inc., Newport, USA), as previously described (6, 21) to continuously release 83 μg DHT/day for 28 days. Sham control animals received identical pellets lacking the steroid. Bone marrow cells were collected from the femur and tibia of 21 days old rats and were cultured and differentiated into macrophages in DMEM/F12 containing recombinant rat macrophage colony-stimulating factor (50 ng/mL; PeproTech, 400-28) for 7 days *in vitro*. Half of the culture media was replaced with fresh media on Day 3. On day 7, macrophages (1.0 × 10^6^ cells) were collected and polarized to M1 and M2, using LPS (10 ρg/mL) + IFN-γ (20 ng/mL; 36 h) and IL-4 (20 ng/ml; 36 h), respectively. In some experiments, macrophages were transfected with miR-379-5p mimic (100 nM) or inhibitor (100 nM; mirVana Life Technologies, Inc.) or scrambled (100 nM) sequence, using Lipofectamine RNAiMAX (Life Technologies, Inc.) for 18 h before treatment *in vitro* (22).

Granulosa cells from preantral follicles (DES-primed 21-day old rats; 1 mg/d, s.c. for 3 consecutive days) and antral follicles (eCG–injected 22-day old rats; 10 IU i.p., animals sacrificed 2 days post-injection) were isolated by follicular puncture (21). Granulosa cells were plated (1 × 10^6^ per well in a 6 well plate) overnight in M199 with 10% FBS under a humidified atmosphere of 95% air and 5% CO2. After culture overnight in serum-free medium, granulosa cells were treated with or without DHT (1 µM; 36 h). DMSO and alcohol were added to the control group (final concentration of 0.001% and 0.005%, respectively) as a vehicle for DHT. Our laboratory has previously examined different concentrations of DHT on various ovarian parameters *in vitro* (23), including follicular growth, granulosa cell proliferation, apoptosis, steroidogenesis and androgen receptor content. We found that 1 μM DHT is the optimal concentration, and hence was used in the current study.

In granulosa cell-macrophage co-culture experiments, granulosa cell-derived exosomal miR-379-5p was tracked by transfecting granulosa cells with alexa-647-labelled miR-379-5p mimic (100 nM; mirVana Life Technologies, Inc.) and GFP-tagged CD63 (System Bioscience; CYTO120-PA-1). Macrophages were imaged by Zeiss LSM880 with AiryScan FAST at the Cell Biology and Image Acquisition at the University of Ottawa.

Conditioned media from rat BMDMs transfected with miR-379-5p mimic (CM-mimic; mirVana mimics, MC10316, Thermo-Fisher) or inhibitor (CM-inhibitor; mirVana inhibitor, MH10316; 18h) were collected and added (1:10 ratio) to preantral and antral follicle granulosa cells, and cultured further for 36 h.

### Immunofluorescence, protein extraction and western blotting

At the time of animal euthanization, the ovaries were fixed in 4% formalin, dehydrated, embedded in paraffin, and cut into 4-µm sections. Sections were de-paraffinized in xylene and rehydrated through a 100-70% ethanol gradient. Antigen retrieval was achieved by boiling the slides in TRIS/EDTA (pH 9.0) or citrate (pH 6.0) buffer in a microwave for 15 min. Sections were blocked with 10% donkey serum in 1% bovine serum albumin in phosphate-buffered saline for 40 min, followed by overnight incubation at 4^o^C with primary antibodies. Primary antibodies, including mouse anti-CD68 (1:100; Abcam) and rabbit anti-CD163 (1:100; Abcam) were diluted in 1% BSA/PBS. Secondary donkey- anti-mouse and donkey-anti-rabbit fluorescent antibodies conjugated with Cy3 and Alexa488 (1:100; Jackson Immunoresearch) incubated for 1 h at room temperature were used to localize or co-localize primary antibodies. The fluorescent dye Hoechst 33342 (1:10,000; Molecular Probes) was used to stain the nuclei. Sections were protected with VectaShield mounting medium (Vector Labs), scanned by Axio Scan.Z1 (Carl Zeiss, Gottingen, Germany) and recorded with the Axion Vision program (Axion Vision software, Zeiss). The macro (FIJI software) was used to apply colour threshold-based selection for the respective signals/channels which then counted the selected particles.

At the end of the culture period, granulosa cells were harvested by trypsin treatment and lysed using Cell Lysis Buffer (Cat#: 9803; Cell Signaling Technology, Inc; Beverly, Massachusetts). Protein extraction and western blotting were performed as described previously (21). Antibodies and their dilutions are summarized in **Supplementary Table 1**. Proteome Profiler Rat XL Cytokine Array (R&D Systems; ARY030) was conducted, as per the manufacturer’s instructions, to compare the content of 79 different cytokines in macrophage culture media. Protein extraction and western blotting were performed as described previously (21).

### Production of recombinant lentiviral particles and injection

The lentiviral gene transfer plasmids pLV-[hsa-miR-379-5p] (Cat.no. miR-p209m) and pLV-[miR-control] (Cat.no. mir-p000) were purchased from the BioSettia Company (San Diego, California, USA); production of recombinant lentiviral particles was performed as per manufacturer’s protocol. Briefly, HEK 293T cells were co-transfected with the transfer vector and the helper plasmids pMD2.G (Addgene) and psPAX2 (Addgene), using the calcium phosphate co-precipitation method. Prior to transfection, 6x10^6^ 293T cells were seeded in 10 cm plates for 24 h in modified Dulbecco’s culture medium containing FBS (10%), penicillin (100 IU/ml), and streptomycin (100 mg/ml) in 5% CO_2_. The culture medium was changed one hour prior to transfection and a total of 18 μg of plasmid DNA was added per dish: [envelope plasmid pMD2.G (3μg), packaging plasmid psPAX2 (6 μg) and transfer vector plasmid (9 μg)]. The precipitate, formed from adding the plasmids to a final volume of 540 μl and 60 μl of 2.5 M CaCl_2_ and then 600 ml of 2x HEPES-buffered saline, was added dropwise immediately to the cultures. The medium was then replaced every 24 h with fresh medium for high-concentration virus production. A high-titered virus was achieved through serial ultracentrifugation, as previously described (24). Briefly, the viral supernatant was collected and filtered through a 0.45 µm filter and transferred into sterilized Ultra-Clear centrifuge tubes (Beckman cat. no. 344058). The viral supernatant was centrifuged (16,500 x g, 90 min, 4°C), pooled, and stored in aliquots at -80°C.

Animals were randomly divided into 2 groups (control and DHT implants) and left and right ovaries in each animal were injected with RFP virus particles and miR-379-5p mimic, respectively, sacrificed 28 days post-injection, and the number and type of macrophages (M1 and M2) at each follicle stage were compared. Viral particles containing RFP or miR-379-5p were injected (2 µl) using a syringe with a glass micro-injection needle (50 μm diameter), as described previously (25).

## Human samples

All human FF and granulosa cell specimens were obtained from the CReATe Biobank, CReATe Fertility Centre with written informed consent. The CReATe Biobank (banking protocols approved by Veritas IRB (Approval#16518), collects biological materials from consenting patients, according to the best practice-based standards of biobanking [7]. Patients undergoing IVF-ICSI have been treated by a routine antagonist protocol, with gonadotropin starting dosing and adjustments based on response to stimulation by the treating physician. Follicles have been collected 35-36hrs following trigger injection. The cumulus-oocyte complex was collected from the aspirate for denudation. The resulting aspirate, containing granulosa cells and follicular fluid, was collected by CReATe BioBank personnel and processed. All samples from the Biobank were approved for use in this study by the Veritas IRB (Approval#16487) and The Ottawa Hospital REB (Protocol #20170453-01H)]. Human ovarian follicular cells and fluids (FF; **Supplementary Table 2**) were collected from lean PCOS (based on Rotterdam criteria; BMI<30) and non-PCOS subjects (premenopausal, 18-40 years) with normal thyroid function and prolactin levels, undergoing assisted reproduction treatment at the CReATe Fertility Centre, Toronto, Canada. Exclusion criteria include (1): known causes of oligomenorrhea other than PCOS; and (2) use of hormone treatment, birth control pill, insulin sensitizers, lipid lowering agents, or medications known to influence insulin sensitivity or serum androgens, within 3 months of the study onset. All FF (PCOS, n = 17 & non-PCOS, n = 20) and follicular cells (PCOS, n = 19 & non-PCOS, n = 19) specimens were obtained from the CReATe Biobank, CReATe Fertility Centre (26–28). Regarding human granulosa cell cultures, each replicate experiment was conducted using the pool of three non-PCOS antral follicle granulosa cell samples (1×10^6^ cells per well; **Supplementary Table 3**). The same follicular fluids used in **Figure 3B** were added to non-PCOS granulosa cells. In this experiment, 200 μl of follicular fluid was added to 2 mL of culture media and a pool of two follicular fluids were used in each experimental replicate. To neutralize galectin-3, neutralizing antibody (Miltenyi Biotec; 130-112-969; 20 μl) was added to follicular fluid (200 μl), incubated 30 min in room temperature and then added to granulosa cell cultures. Isotype control antibody, rat IgG2a (Miltenyi Biotec; 130-102-652) was added to PCOS and non-PCOS experimental groups. Human recombinant galectin-3 (R & D systems; n = 3; 0.03 ng/mL) was added to non-PCOS granulosa cell cultures.

### Flow cytometry, multiplex magnetic bead immunoassay and ELISA assays

Cleared follicular fluid samples (n=17 PCOS, n=20 non-PCOS) were diluted (1:2) with Calibrator Diluent and distributed into a 96 well plate. Samples were assayed utilizing immunoassay, according to the manufacturer’s instructions (Luminex Assay, R&D Systems, Oakville, ON). All samples were assayed in technical duplicates. The following analytes were assessed using a Luminex Assay: Cystatin C, Galectin-1, Galectin-3, IL6, and Osteoprotegerin. Briefly, the analytes were captured using specific immunoglobulin-coated magnetic beads, while a biotin-conjugated antibody was added to provide analyte-specific quantitative readout, and a fluorophore-conjugated (PE) detection antibody was added to amplify the signal to detection range. Washes were conducted between additions to remove any unbound molecules. Finally, the beads were resuspended in wash buffer and analyzed using the MACSQuant Analyzer flow cytometer (Miltenyi Biotec, Germany). Events corresponding to beads were selected based on forward scatter (FSC) and side scatter (SSC). Specific single analyte bead populations were identified and selected using APC and PE-Vio770 filters (Classification Fluorescence). Each individual population was then assessed using the PE filter to determine the median fluorescence intensity (MFI) of the population. The MFI of the standards were plotted against the known concentration of the standards and a standard curve was constructed. Utilizing linear regression, concentrations were determined for each analyte.

Rat and human granulosa cells were fixed, permeabilized and stained for Ki67. Regarding macrophage polarization assessment, human follicular cells were fixed, permeabilized and stained for macrophage pan marker (CD68) and polarization markers (human M1: CD68+HLA-DR+ and M2: CD68+CD206+). Rat BMDMs were also fixed and stained for the macrophage polarization markers, M1: CD86+ and M2: CD163+. Flow cytometric acquisition was performed and analyzed as described previously (6).

Concentration of TNF-α was measured by a rat TNF-α ELISA kit (Abcam, ab100785) in 100 µl of CM-mimic and CM-inhibitor. All ELISA measurements were carried out according to the manufacturer’s instructions. Optical densities (OD) were determined at 450 nm and compared to a standard curve, using a microtiter plate reader. The blank was subtracted from the triplicate readings for each standard and test sample.

### Statistics

T-tests, and one-, two-, or three-way analyses of variance (ANOVA) were used to assess the effects of, and interactions between variables. This was followed by multiple comparison analysis with Tukey post hoc test, using Prism v.7 (GraphPad, San Diego, CA) and Sigma plot v.12 (Systat Software, San Jose, CA). P < 0.05 was considered statistically significant.

SPSS (v 25) was used to assess the sensitivity and specificity of FF galectin-1 and -3 content alone or in combination with anti-Müllerian hormone (AMH) in prediction of PCOS. Significant variables were entered into a forward logistic regression predicting PCOS status. Forward regression was used to preserve power with multiple predictors, avoid multicollinearity, and develop the most parsimonious model possible (29). Final models were then used to develop ROC curves comparing the final logisitc regressions models to PCOS status. ROC curves were compared using Medcalc (v. 19.5.6). Outlier analysis was undertaken visually and through the use of standardized z scores, and normality was examined visually as well as through skew and kurtosis analysis (29). Multicollinearity was examined in all models.

## Results

**Figure 1:**
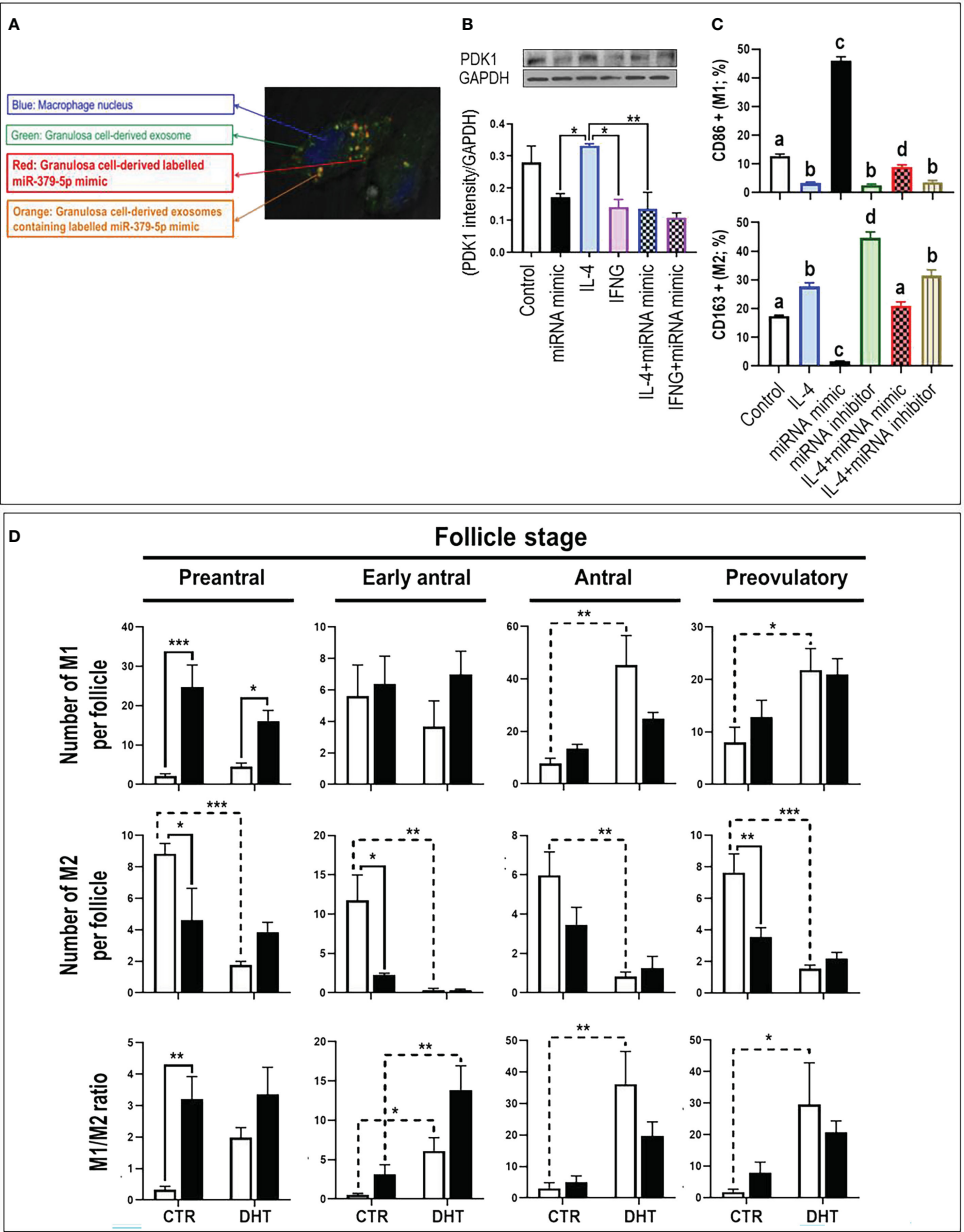
MiR-379-5p (miR379) increases M1/M2 ratio in preantral follicle stage by targeting PDK1. **(A)** Granulosa cells were transfected with CD63-GFP (green) and Alexa-647 labelled miR-379-5p (red), allowing the tracking of exosome and miR-379-5p in co-culture system. Tracking granulosa cell derived exosomes (green; GFP) and miR-379-5p (red; alexa-647) signals indicate that macrophages engulfs granulosa cell derived exosomes containing labeled miR-379-5p (orange). **(B)** IL4-induced M2 had greater PDK1 content compared to IFNγ-induced M1. Pre-treatment of bone marrow derived macrophages with miR379 mimic alone or in combination with IL4 reduced PDK1 to a similar level of IFNγ-M1. **(C)** miR379 polarizes macrophages towards M1. Transfection of macrophages with miR379 mimic polarized M0 to M1, a mechanism which was reversed by its inhibitor. **(D)** The ovarian miR379 overexpression increased M1 population in preantral follicles of both control and DHT rats. MiR379 overexpression significantly reduced M2 population in preantral, early antral and preovulatory follicles, increasing the M1/M2 ratio in control preantral follicles. Androgen excess increased M1 population in antral and preovulatory follicles, but reduced M2 population in all stages. The ratio of M1/M2 increased in early antral, antral and preovulatory follicles in response to androgen; Results are expressed as mean±SEM (n=3 replicates each with 2 rats/group). Data were analyzed by one-way ANOVA & tukey post hoc. *P<0.05; **P<0.01; ***P<0.001; **(C)** Different letters indicate significant differences between groups (P<0.05). To produce bone marrow derived macrophage, bone marrow cells were isolated from rat femur and tibia and differentiated to macrophages with M-CSF for 7 days in vitro. Granulosa cells were isolated from preantral follicles (diethylstilbestrol-primed immature rats; day 21, 1 mg/d, subcutaneously, for 3 consecutive days) and transfected with miR-379-5p mimic (mirVana mimics, MC10316, Thermo-Fisher; 18h) and were further cultured for 36h. Macrophages were polarized to M1 and M2 by LPS (10 ρg/mL) + IFN-γ (20 ng/mL) and IL-4 (20ng/mL) treatment for 36h, respectively.

Macrophages uptake GC-derived exosomal miR379. To confirm whether macrophages take up GC-derived exosomes, GCs were co-cultured with macrophages using Transwell^TM^ dishes. To track GC-derived exosomes and miR379 in co-culture system, GCs were transfected with CD63 (exosome marker)-GFP (green) and Alexa-647 labelled miR379 (red) (**Supplementary Figure S1**). Tracking GC-derived exosomes and miR379 signals indicated that macrophages engulf GC-derived exosomal labeled miR379 (orange) (**Figure 1A**).

MiR379 polarizes macrophages to M1 by targeting PDK1. To determine if PDK1 is involved in macrophage polarization, M0 macrophages were polarized to M1 and M2 by culturing with LPS+IFNγ and IL4, respectively, and PDK1 content was assessed (**Figure 1B**). Our results indicate that IL-4-induced M2 had greater PDK1 content compared to LPS+IFN-γ-induced M1, implicating PDK1 in macrophage polarization. To further investigate the role of miR379 in macrophage polarization, BMDMs were pretreated with miR379 mimic and cellular PDK1 content was assessed. Pretreatment of BMDMs with miR379 mimic alone or in combination with IL-4-induced M2 reduced PDK1 to a similar level of LPS+IFN-γ-M1 (**Figure 1B**). MiR379 targets PDK1 and inhibits the AKT pathway, a signaling cascade which plays a significant role in M2 polarization. Therefore, to further investigate the role of miR379 in macrophage polarization, BMDMs (M0) and M2 macrophages were transfected with miR379 mimic or inhibitor and macrophage polarization was assessed. Our flow cytometry results showed that transfection of macrophages with miR379 mimic polarized M0 to M1 (as evidenced by an increase in CD86 positivity), whereas its inhibitor polarized M0 to M2 (as evidenced by an increase in CD163 staining), suggesting that miR379 induces M1 polarization, possibly through targeting PDK1 (**Figure 1C**).

MiR379 increases M1/M2 ratio in preantral follicle stage. To determine the role of miR379 in macrophage polarization *in vivo* and whether miR379 overexpression could have a synergic effect with androgens in androgenized rats, lentiviral miR379 mimic was introduced under the ovarian bursa and ovarian macrophage population and polarization were compared in the following treatments: lentivirus-RFP (control), lentivirus-RFP-miR379, DHT+lentivirus-RFP, DHT+lentivirus-RFP-miR379 (**Supplementary Figure S2**, **Figure 1D**). Our results indicated that ovarian miR379 overexpression reduced the M2 population in preantral, early antral and preovulatory follicles of control rats and increased M1 macrophages in preantral follicles of control and DHT rats. This resulted in an increased M1/M2 ratio in the preantral follicles of control rats only. On the other hand, androgen excess increased the M1 population in both antral and preovulatory follicles, but reduced the M2 population in all follicular stages. These results suggest that miR379 exerts its immune-modulatory effect mainly at the preantral follicle stage (**Figure 1C**).

**Figure 2:**
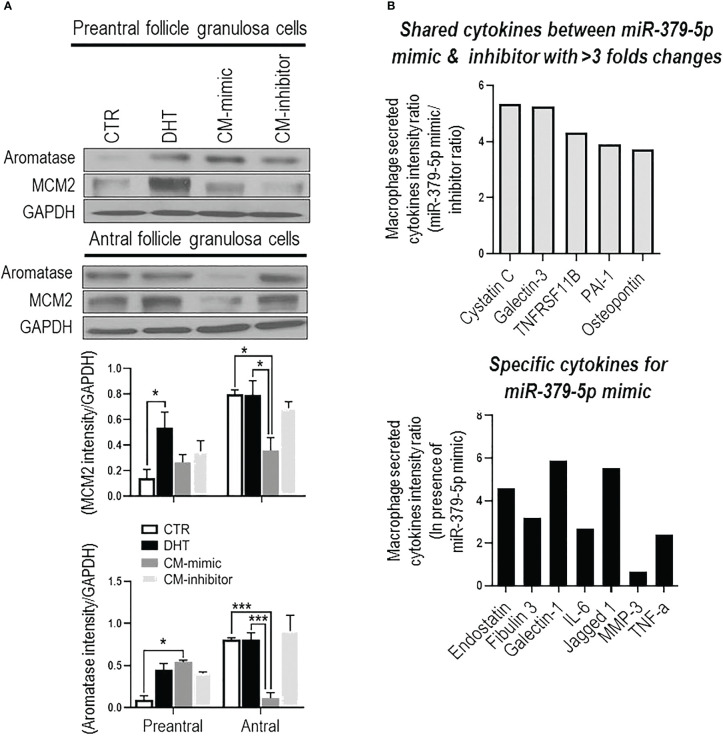
MiR-379-5p-induced M1 polarization regulates granulosa cell proliferation in a follicular stage-specific manner by increasing pro-inflammatory cytokine level. **(A)** Conditioned media from macrophages transfected by miR379 mimic (CM-mimic) increases aromatase content without affecting proliferation in preantral follicle GC, while it reduces aromatase and proliferation in that of antral follicles. The aromatase and proliferative responses were not significantly influenced by the presence of the CM-inhibitor compared to control in both follicle stages; **(B)** Profile of cytokines (Proteome Profiler Rat XL Cytokine Array; R&D Systems) in macrophage condition medium indicates that CM-mimic had higher content of inflammatory cytokines, including Cystatin C (5.4 fold), Galectin-3 (5.3 fold), TNFRS11B (4.3 fold), PAI-1 (3.9 fold) and Osteopontin (3.7 fold). In addition, Endostatin, Fibulin 3, TNF-α, Galectin-1, IL-6, Jagged 1 and MMP3 were specific cytokines to CM-mimic. Results are expressed as mean±SEM (n=3 replicates each with 2 rats/group). Data were analyzed by two-way ANOVA and tukey post hoc. *P<0.05, ***P<0.001.

MiR379-induced M1 polarization regulates GC proliferation, and aromatase content and is follicular stage-dependent. To determine if miR379-induced M1 polarization alters GC proliferation and aromatase content, and if this is follicular stage-dependent, conditioned media from BMDM transfected with miR379 mimic (CM-mimic), miR379 inhibitor (CM-inhibitor) or DHT were added to GC cultures of preantral and antral follicles (**Figure 2A**); and MCM2 (proliferation) and aromatase contents were assessed. CM-mimic increased aromatase protein content (p<0.05) without significantly affecting proliferation in preantral follicle GCs (**Figure 2A**), while it reduced aromatase levels and proliferation of GCs from antral follicles (**Figure 2A**). The aromatase and proliferative responses were not significantly influenced by the presence of the CM-inhibitor compared to control in both follicle stages. Taken together, these results suggest that miR379-induced M1 polarization suppresses GC proliferation and aromatase content specifically at the antral follicle stage.

MiR379-induced M1 polarization increases the levels of inflammatory cytokines in conditioned media. To investigate the role of cytokines in GC proliferation and aromatase content, CM-mimic and CM-inhibitor cytokines were first profiled by the Proteome Profiler Cytokine Array. Our results indicate that CM-mimic mainly contained inflammatory cytokines, including Cystatin C, galectin-1, galectin-3, TNFRS11B, PAI-1 and Osteopontin, compared to CM-inhibitor (**Figure 2B**). These are shared cytokines between CM-mimic and CM-inhibitor with > 3-fold changes in their signal intensity ratio. We further validated the Proteome Profiler Cytokine Array results by assessing TNF-α in CM-mimic and CM-inhibitor using an ELISA. Our results confirmed that TNF-α is a specific cytokine in CM-mimic (**Supplementary Figure S3**).

**Figure 3:**
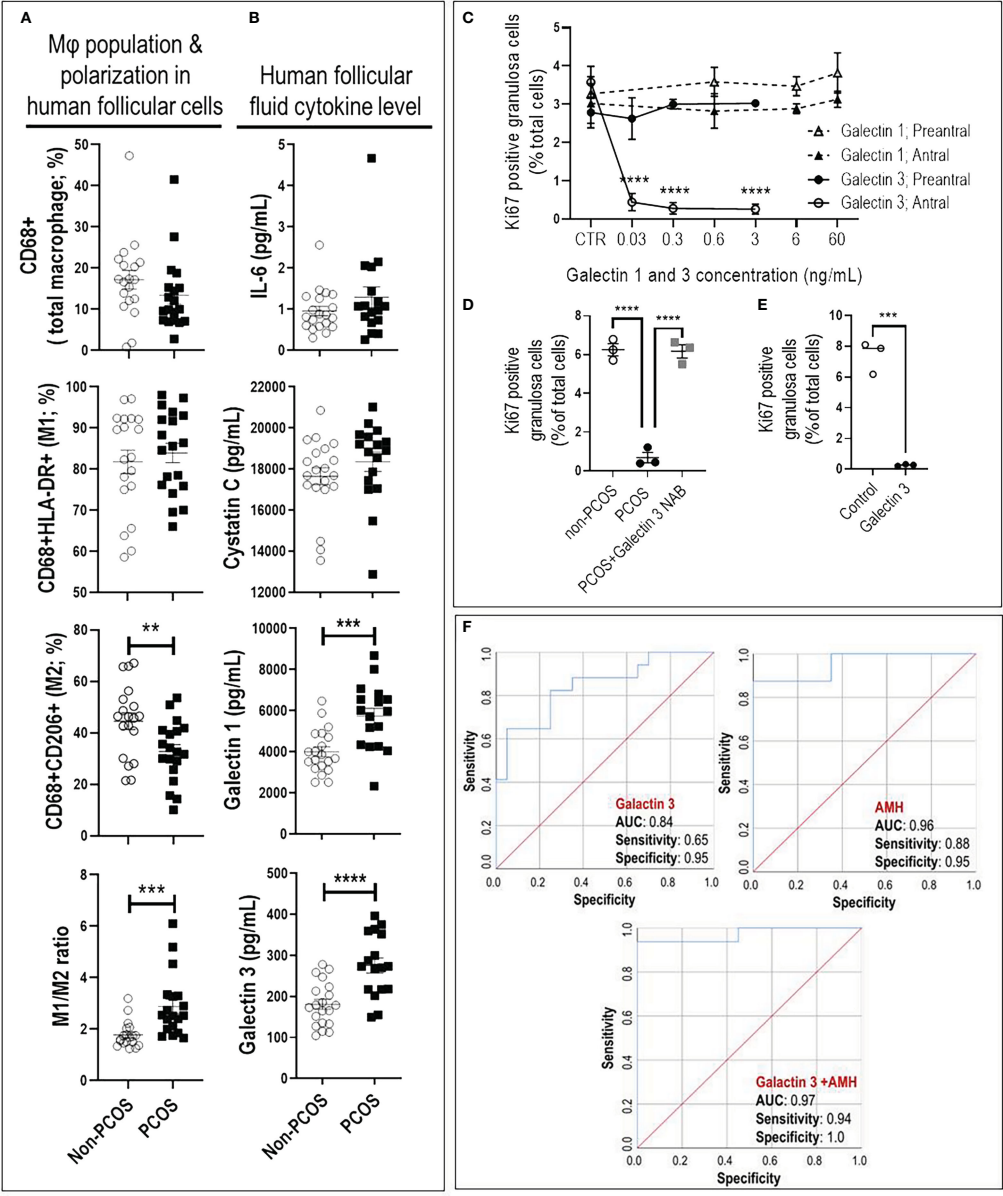
Increased M1/M2 ratio and galectin-3 suppresses granulosa cell proliferation in antral, but not preantral follicle stage, as evident in PCOS. **(A)** PCOS subjects had lower M2 population and higher M1/M2 ratio compared to non-PCOS; **(B)** PCOS subjects had higher FF content of galectin-1 and -3 compared to non-PCOS; **(C)** Galectin-1 did not affect rat GC proliferation in preantral and antral follicles, galectin-3 significantly reduced GC proliferation in antral but not preantral follicles; **(D)** Adding PCOS FF to non-PCOS GCs significantly reduced cell proliferation compared to those treated with non-PCOS FF. However, the effect of PCOS FF on GC proliferation was blocked using galectin-3 neutralizing antibody; **(E)** Adding human recombinant galectin-3 to non-PCOS GCs significantly suppress proliferation; **(F)** The combination of galectin-3 and Anti-Müllerian hormone (AMH) improve sensitivity and specificity of PCOS prediction compared to AMH. AMH: AUC=0.96 with sensitivity=0.88 and specificity=0.95 (p<0.0001); Galectin-3: AUC=0.84 with sensitivity=0.65 and specificity=0.95 (p<0.001); Galectin-3+AMH: AUC=0.977 with sensitivity=0.94 and specificity=1 (p<0.0001). Results are expressed as mean±SEM (n=3 replicates each with 2 rats/group). Data were analyzed by two-way ANOVA and tukey post hoc. **P<0.01, ***P<0.001, ****P<0.0001.

Increased M1/M2 ratio and inflammatory cytokines (galectin-1 and 3) indicate a pro-inflammatory condition in ovaries of PCOS subjects. Follicular macrophage population and polarization were assessed to examine their association with human antral follicle GCs from PCOS and non-PCOS subjects (**Figure 3**). PCOS subjects had a significantly lower M2 population (p<0.01), resulting in a higher M1/M2 ratio (p<0.001), compared to non-PCOS subjects (**Figure 3A**).

To further investigate if an increased M1/M2 ratio in PCOS subjects is associated with higher inflammatory cytokines in follicular fluid (FF), the concentration of galectin-1, galectin-3, Osteoprotegerin, IL6 and Cystatin C were compared between FF from PCOS and non-PCOS subjects. Our results indicated that FF content of galectin-1 and -3 were significantly higher in PCOS subjects compared to the non-PCOS subjects; however, there was no difference in other cytokines (**Figure 3B**, **Supplementary Table 2**).

Galectin-3 suppresses GC proliferation in antral, but not preantral follicles. To determine if reduced antral follicle GC proliferation in response to CM-mimic is as a result of galectin-1 or galectin-3 action; and whether this response is follicular stage-dependent, rat preantral and antral GC were cultured with galectin-1 (0, 0.6, 6 and 60 ng/mL) and galectin-3 (0, 0.03, 0.3 and 3 ng/mL), and their proliferation were assessed based on the changes in Ki67-positive cells (flow cytometry). The concentrations of galectin-1 and galectin-3 for *in vitro* studies were selected based on their average FF concentration in human PCOS subjects; i.e. 6 and 0.3 ng/mL, respectively. Our results demonstrated that while galectin-1 did not affect GC proliferation in both preantral and antral follicles, galectin-3 significantly reduced GC proliferation in antral, but not preantral follicles (**Figure 3C**).

Our previous studies indicate that antral follicle GC from PCOS subjects exhibit lower proliferation compared to those of non-PCOS (17). Here, to further investigate if reduced antral follicle GC in human PCOS subjects could be a result of higher galectin-3, non-PCOS GCs (**Supplementary Table 3**) were treated with FF of non-PCOS and PCOS subjects (1:10 dilution) and cell proliferation was assessed by Ki67 staining. Treatment of GC with PCOS FF significantly suppressed proliferation compared to that of non-PCOS, a response reversible by neutralization of galectin-3 in PCOS FF (**Figure 3D**). To demonstrate if human GC proliferation could be down-regulated by galectin-3, antral follicles GCs from non-PCOS subjects were treated with recombinant galectin-3 protein. Flow cytometry results indicate that galectin-3 significantly reduced antral follicle GC proliferation, as evident by a marked decrease in Ki67-positive cells (**Figure 3E**).

The combination of galectin-3 and AMH improves sensitivity and specificity of PCOS prediction. To determine if galectin-1 and galectin-3 can be used as predictors for PCOS, we first compared potential predictors using univariate analysis between PCOS and non-PCOS subjects, including Age, BMI, LH, Trigger E2, AMH, galectin-1, galectin-3, Cystatin C, Osteoprotegerin and IL6. Our results indicated that AMH, galectin-1 and galectin-3 were significantly increased in PCOS compared to non-PCOS subjects; therefore, they could play as potential predictors for PCOS status (**Figure 3B**, **Supplementary Table 2**). To identify the most precise explanatory model, AMH, galectin-1 and galectin-3 were entered into a forward entry binary logistic regression model. In the final model, AMH was identified (*P*= 0.04) as the only predictor for PCOS status, explaining 65% of the variance in PCOS diagnosis. There was a significant correlation found between AMH and both galectin-1 (R=0.33; *P*=0.04) and galectin-3 levels (R=0.48; *P*=0.003), indicating that AMH may mitigate the importance of galectin-1 and galectin-3 as a predictor of PCOS. To further explore the predictive power of galectin-1 and galectin-3, AMH was excluded from the model, and galectin-1 and -3 were retained. In this model, galectin-3 remained in model (*P*=0.003) and explained 36% of the variance in PCOS diagnosis. As the final step, to investigate the test performance of galectin-3 vs AMH (**Figure 3F**), three ROC curves were constructed: AMH, galectin-3, and galectin-3+AMH. The AMH model was significant (*P* < 0.0001), with an area under the curve (AUC) of 0.96, and it also outperformed the galectin-3 only model (*P* < 0.001, AUC=0.84). Although the combination of galectin-3+AMH did not change the AUC (0.97), it improved the model sensitivity and specificity, 0.94 and 1.00, respectively (**Figure 3F**).

## Discussion

Exosomes play important roles in intercellular communication by serving as vehicles for transferring cellular constituents such as proteins, lipids and nucleic acids. However, very little is known about the role of exosome release in determining the cellular miRNA content and function in exosome-receiving cells. We have recently found that exosome release serves as a proliferative mechanism to regulate the cellular miR-379-5p (miR379) content in preantral follicle development in response to androgen stimulation (17). More specifically, androgen induces exosomal miR379 release in GC from preantral, but not antral follicles. Reduced preantral follicle GC miR379 content increases PDK1 content and enhances their proliferation (17). In this study, we have demonstrated that uptake of GC-derived exosomes containing miR379 by macrophages inhibits PDK1 and shifts macrophage polarization to M1. Similarly, deletion of PTEN increased the levels of Arg1 and M2 polarization, indicating a major role of the AKT pathway in anti-inflammatory macrophage polarization (18, 19). Moreover, inhibition of AKT phosphorylation through knockdown of PDK1 increased M1 polarization and increased the susceptibility of mice to endotoxin shock (20). These results suggest that the regulation of PDK1-AKT signaling is a central node for controlling M2 polarization and increased macrophage miR379 content through uptake of GC-derived exosomal miR379 inhibits PDK1-mediated M2 polarization.

Macrophage polarization is very important in GC proliferation, ovarian follicular development and ovulation (6). Studies with androgenized rats and human PCOS subjects have demonstrated that androgen suppresses ovarian M2 polarization and significantly increases the M1/M2 ratio in antral follicles (6). Similarly, in this study androgen significantly increased the M1/M2 ratio in early antral, antral and preovulatory follicles. On the other hand, ovarian overexpression of miR379 inhibited M2, increased M1 and the M1/M2 ratio in preantral follicles sepcifically. These results support our previous findings where androgen induced specific exosome packaging and release of miR379 from preantral follicle GCs; as well as promoting uptake of miR379 enriched exosomes. This suppressed androgen-induced proliferation by increasing miR379 cellular content in preantral follicle GCs (17). These results suggest that uptake of GC derived miR379 enriched exosomes inhibits PDK1 in macrophages; a process that polarizes them to M1.

Galectins are a family of evolutionarily conserved carbohydrate-binding proteins (30, 31) involved in cell activation, differentiation, proliferation, migration and apoptosis (32–35). Culture media from macrophages treated with miR379 mimic and FF from PCOS subjects exhibited a significantly higher galectin-1 and galectin-3 content. Further investigation on the influence of galectins on GC proliferation indicates that galectin-3, but not galectin-1, suppressed antral follicle GC proliferation. This response appeared to be follicle stage specific as no significant reduction in preantral follicle GC proliferation was observed under the same conditions. Histological assessment of ovarian sections demonstrates that macrophages and apoptotic GCs are the main source of galectin-3 (36–38). Galectin-3 is pro-inflammatory cytokine and deletion of galectin-3 in mice reduced inflammation, number of macrophages and IL1β production (39). Galectin-3 has both anti- and pro-apoptotic effects (40). Studies on human thymocytes and T cells demonstrate that, in contrast to the anti-apoptotic function of intracellular galectin-3, extracellular galectin-3 directly induces apoptosis in human thymocytes and T cells through CD71 (40). Therefore, our results demonstrate, for first time, that macrophage-derived galectin-3 suppresses GC proliferation in follicle stage-dependent manner. Further studies are required to determine the precise cellular mechanisms involved.

AMH is produced by GCs from follicles that are < 8 mm (41, 42) and regulates early follicular recruitment (43). PCOS subjects have a higher serum concentration of AMH (44, 45) and AMH correlates with oligoamenorrhea and hyperandrogenism (46–50), AMH has been proposed as a biomarker for PCOS (51). Serum level of galectin-3 is significantly higher in PCOS subjects (52, 53), is associated with insulin resistance (52, 54) and has been proposed as a biomarker for detecting prediabetes, diabetes (55) and inflammation (56). In the present study we found that FF from PCOS subjects had higher galectin-3 content and in combination with AMH improves the sensitivity and specificity of PCOS prediction compared to AMH alone. However, further studies are needed to determine how galectin-3 regulates follicular growth and ovarian function.

**Figure 4:**
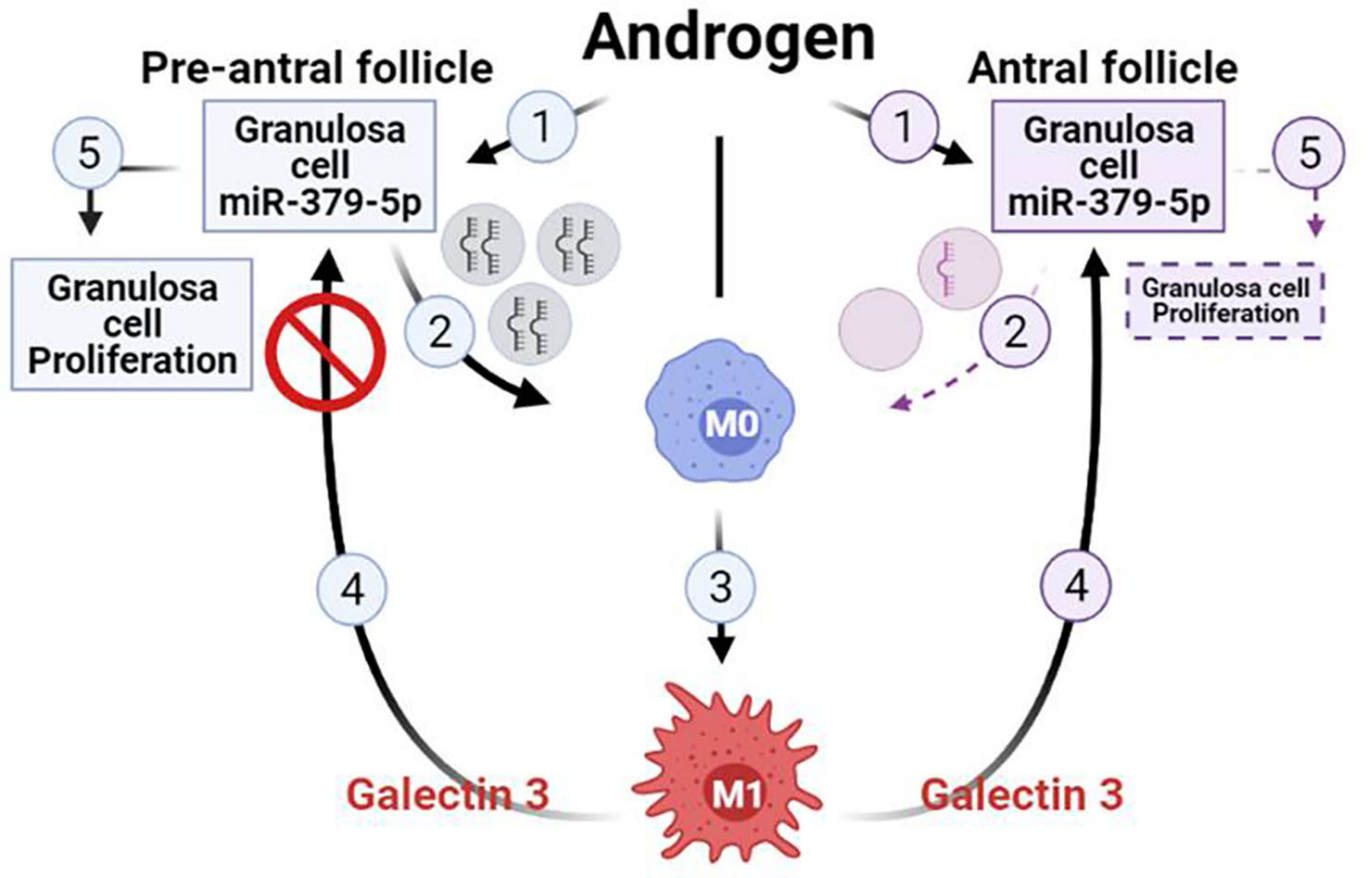
Hypothetical model illustrating that uptake of GC-derived exosomal miR-379-5p by macrophages increases M1 and macrophage release of galectin-3, a response which inhibits GC proliferation in antral but not preantral follicles. Androgen induces GC exosomal miR379 release from preantral but not antral follicles. The uptake of GC-derived exosomal miR379 suppresses PDK1 in macrophages, a cellular mechanism shifting macrophage polarization towards M1. M1 macrophage secretes higher level of galectin-3 in FF, a inflammatory cytokine that specifically suppressed GC proliferation in antral but not preantral follicles. (Created in BioRender.com).

In conclusion, our findings suggest that miR379 inhibits M2 macrophage polarization, increases the M1/M2 ratio, as well as macrophage secretion of galectin-3. These responses inhibit GC proliferation in antral, but not preantral follicles. This phenomenon is likely initiated through the release of granulosa cell-derived exosomal miR379 in a follicular-stage dependent manner. Therefore, we propose a hypothetical model to facilitate future investigation into the role of GC-derived exosomal miR379 in the androgenic control of macrophage polarization and subsequently GC proliferation (**Figure 4**). Androgen induces GC exosomal miR379 release from preantral but not antral follicles. The uptake of GC derived exosomal miR379 suppresses PDK1 in macrophages, shifting them to M1. M1 macrophage secretes higher levels of galectin-3 in FF; an inflammatory cytokine that specifically suppresses GC proliferation in antral, but not preantral follicles (**Figure 4**). In this study, we demonstrated that androgen-induced miR379 regulates GC proliferation by macrophage-derived galectin-3 in a follicle stage-dependent manner. However, further studies are required to determine 1) how galectin-3 regulates follicular growth and ovarian function; 2) if, and how, obesity alters galectin-3 in PCOS subjects; and 3) whether and how metformin could reduce the galectin-3 levels in PCOS subjects.

## Data availability statement

The raw data supporting the conclusions of this article will be made available by the authors, without undue reservation.

## Ethics statement

All human FF and granulosa cell specimens were obtained from the CReATe Biobank, CReATe Fertility Centre with written informed consent. The CReATe Biobank [banking protocols approved by Veritas IRB (Approval#16518), collects biological materials from consenting patients, according to the best practice-based standards of biobanking. All samples from the Biobank were approved for use in this study by the Veritas IRB (Approval#16487) and The Ottawa Hospital REB (Protocol #20170453-01H)]. The patients/participants provided their written informed consent to participate in this study. All animal procedures were carried out in accordance with the Guidelines for the Care and Use of Laboratory Animals, Canadian Council on Animal Care, and were approved by the University of Ottawa Animal Care Committee.

## Author contributions

RS and BT designed the experiments. RS, AA, BW, MA-W, BP and AG performed the experiments. PS, SJ and CL provided human samples. KB, DB, BV, JL contributed analytic tools. RS analyzed experimental data. RS prepared the manuscript with input from BT, BV, DB, KB, JL, and CL. All authors contributed to the article and approved the submitted version. BT and CL provided financial support for the project.
