# Supplementary material for: Granulosa cell-derived miR-379-5p regulates macrophage polarization in polycystic ovarian syndrome
Source: Front Immunol. 2023 Mar 24;14:1104550. doi: 10.3389/fimmu.2023.1104550 (PMC10081157; doi:10.3389/fimmu.2023.1104550)
Supplement: Supplementary file 1 [file Presentation_1.pptx]

## Slide 1
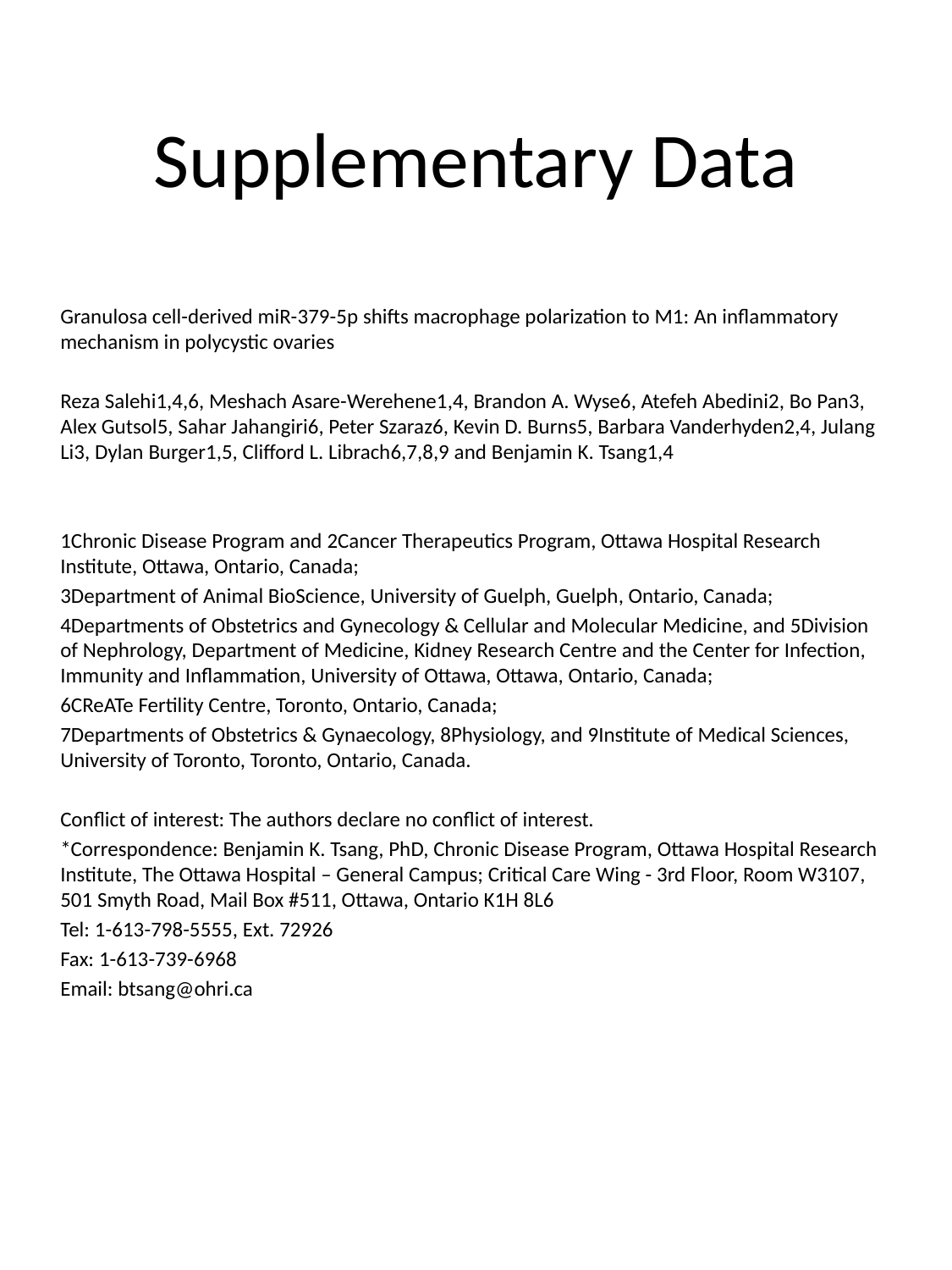

# Supplementary Data
Granulosa cell-derived miR-379-5p shifts macrophage polarization to M1: An inflammatory mechanism in polycystic ovaries
Reza Salehi1,4,6, Meshach Asare-Werehene1,4, Brandon A. Wyse6, Atefeh Abedini2, Bo Pan3, Alex Gutsol5, Sahar Jahangiri6, Peter Szaraz6, Kevin D. Burns5, Barbara Vanderhyden2,4, Julang Li3, Dylan Burger1,5, Clifford L. Librach6,7,8,9 and Benjamin K. Tsang1,4
1Chronic Disease Program and 2Cancer Therapeutics Program, Ottawa Hospital Research Institute, Ottawa, Ontario, Canada;
3Department of Animal BioScience, University of Guelph, Guelph, Ontario, Canada;
4Departments of Obstetrics and Gynecology & Cellular and Molecular Medicine, and 5Division of Nephrology, Department of Medicine, Kidney Research Centre and the Center for Infection, Immunity and Inflammation, University of Ottawa, Ottawa, Ontario, Canada;
6CReATe Fertility Centre, Toronto, Ontario, Canada;
7Departments of Obstetrics & Gynaecology, 8Physiology, and 9Institute of Medical Sciences, University of Toronto, Toronto, Ontario, Canada.
Conflict of interest: The authors declare no conflict of interest.
*Correspondence: Benjamin K. Tsang, PhD, Chronic Disease Program, Ottawa Hospital Research Institute, The Ottawa Hospital – General Campus; Critical Care Wing - 3rd Floor, Room W3107, 501 Smyth Road, Mail Box #511, Ottawa, Ontario K1H 8L6
Tel: 1-613-798-5555, Ext. 72926
Fax: 1-613-739-6968
Email: btsang@ohri.ca

## Slide 2
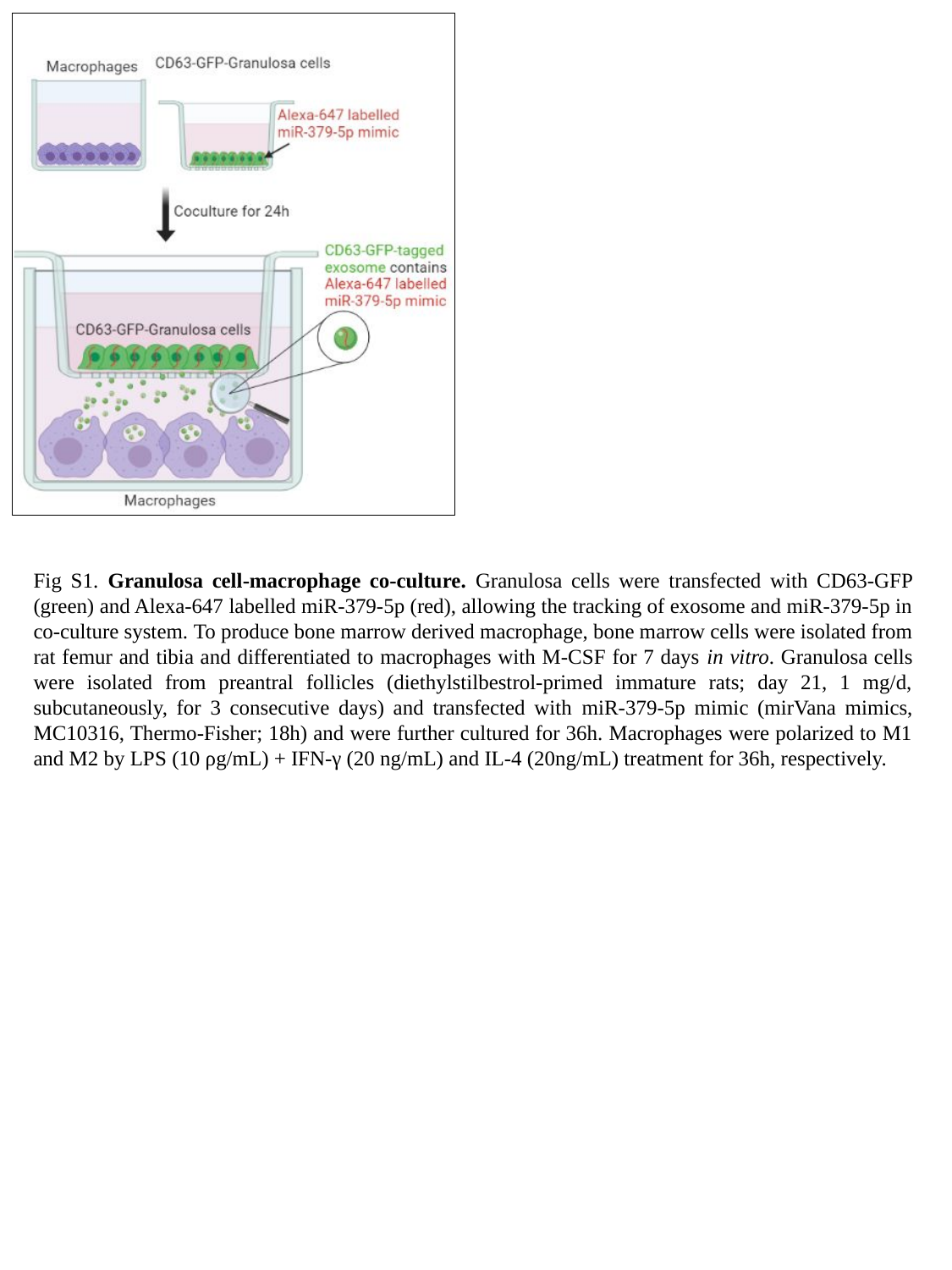

Fig S1. Granulosa cell-macrophage co-culture. Granulosa cells were transfected with CD63-GFP (green) and Alexa-647 labelled miR-379-5p (red), allowing the tracking of exosome and miR-379-5p in co-culture system. To produce bone marrow derived macrophage, bone marrow cells were isolated from rat femur and tibia and differentiated to macrophages with M-CSF for 7 days in vitro. Granulosa cells were isolated from preantral follicles (diethylstilbestrol-primed immature rats; day 21, 1 mg/d, subcutaneously, for 3 consecutive days) and transfected with miR-379-5p mimic (mirVana mimics, MC10316, Thermo-Fisher; 18h) and were further cultured for 36h. Macrophages were polarized to M1 and M2 by LPS (10 ρg/mL) + IFN-γ (20 ng/mL) and IL-4 (20ng/mL) treatment for 36h, respectively.

## Slide 3
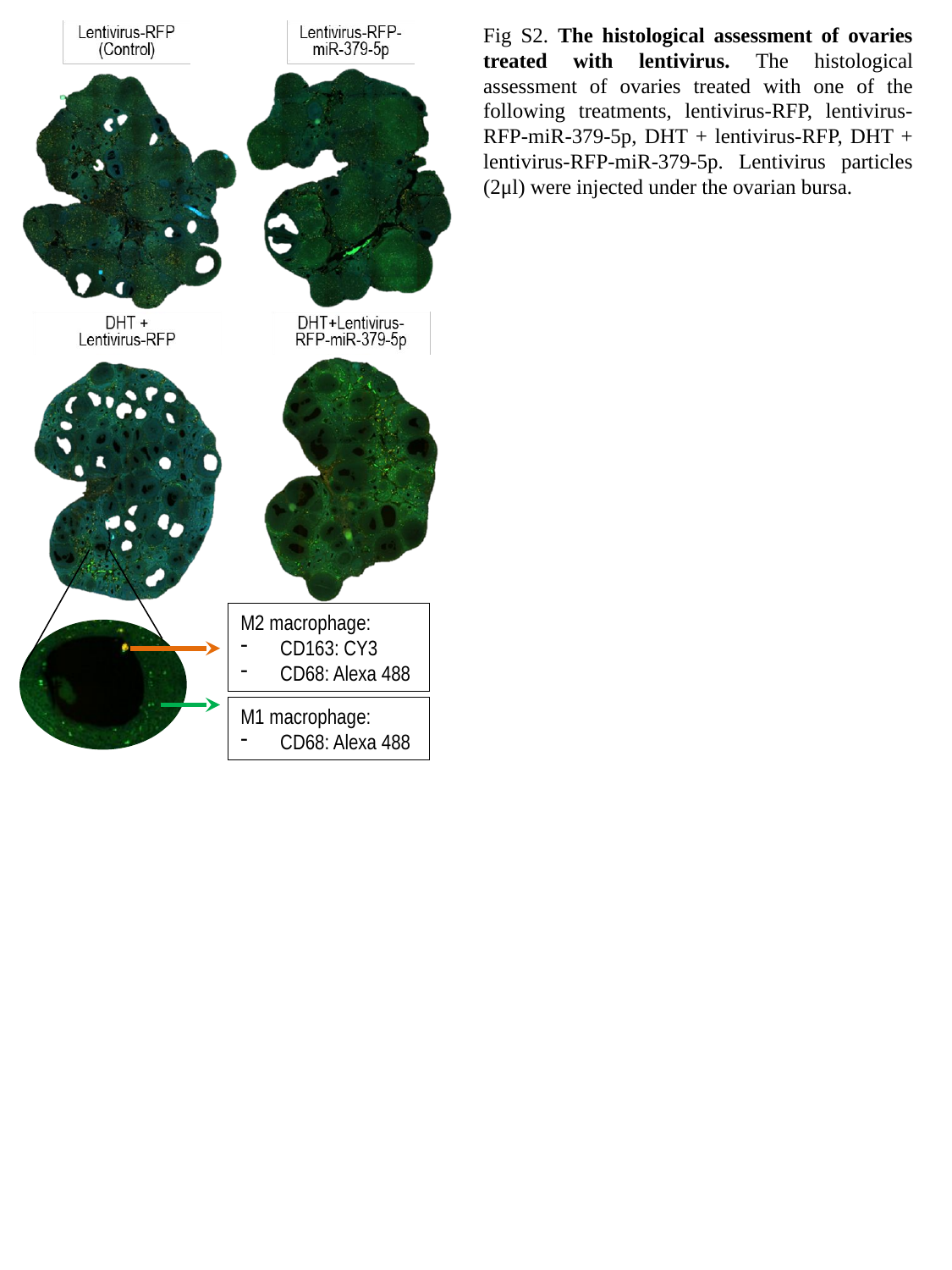

Fig S2. The histological assessment of ovaries treated with lentivirus. The histological assessment of ovaries treated with one of the following treatments, lentivirus-RFP, lentivirus-RFP-miR-379-5p, DHT + lentivirus-RFP, DHT + lentivirus-RFP-miR-379-5p. Lentivirus particles (2μl) were injected under the ovarian bursa.
M2 macrophage:
CD163: CY3
CD68: Alexa 488
M1 macrophage:
CD68: Alexa 488

## Slide 4
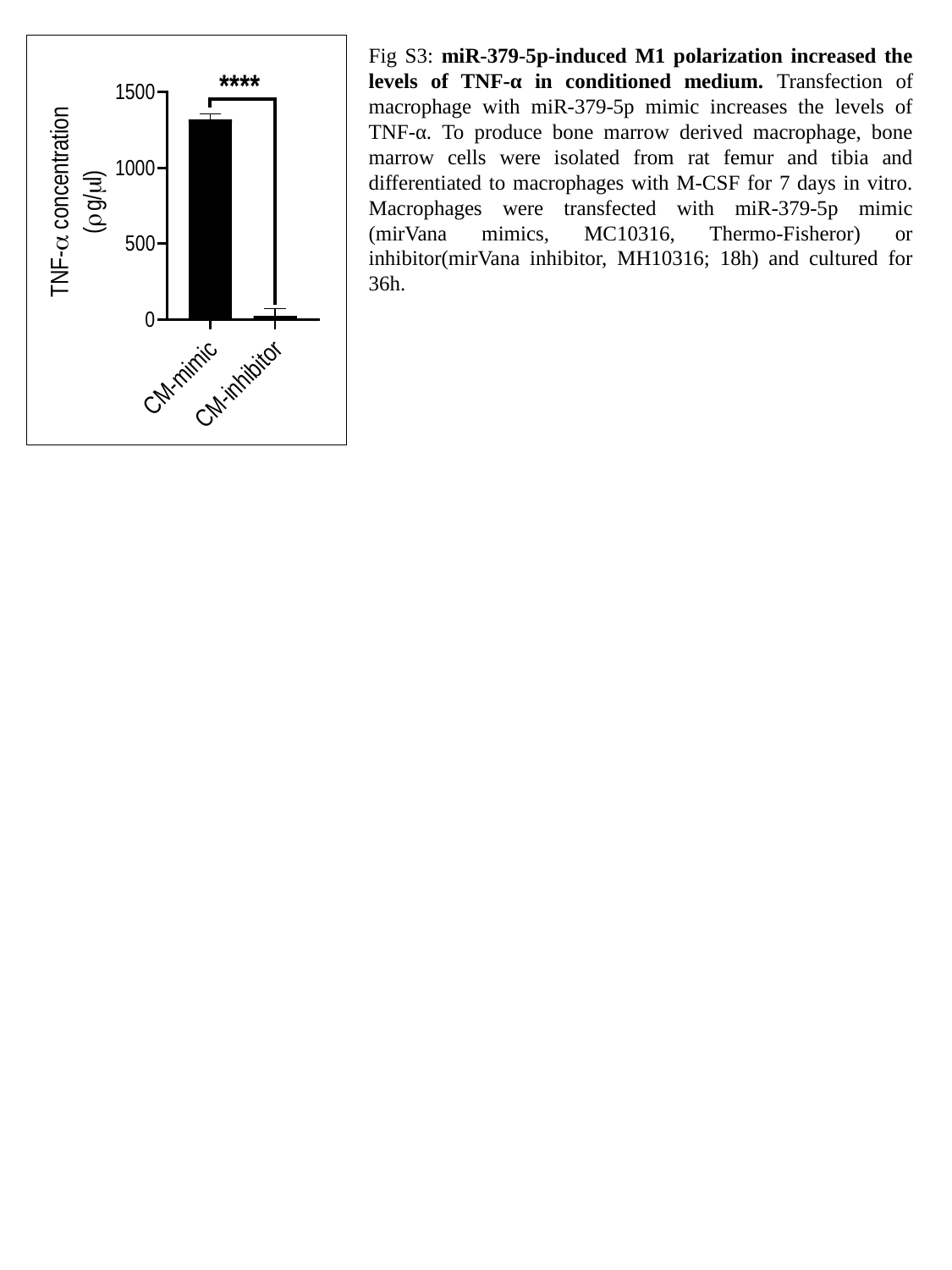

Fig S3: miR-379-5p-induced M1 polarization increased the levels of TNF-α in conditioned medium. Transfection of macrophage with miR-379-5p mimic increases the levels of TNF-α. To produce bone marrow derived macrophage, bone marrow cells were isolated from rat femur and tibia and differentiated to macrophages with M-CSF for 7 days in vitro. Macrophages were transfected with miR-379-5p mimic (mirVana mimics, MC10316, Thermo-Fisheror) or inhibitor(mirVana inhibitor, MH10316; 18h) and cultured for 36h.

## Slide 5
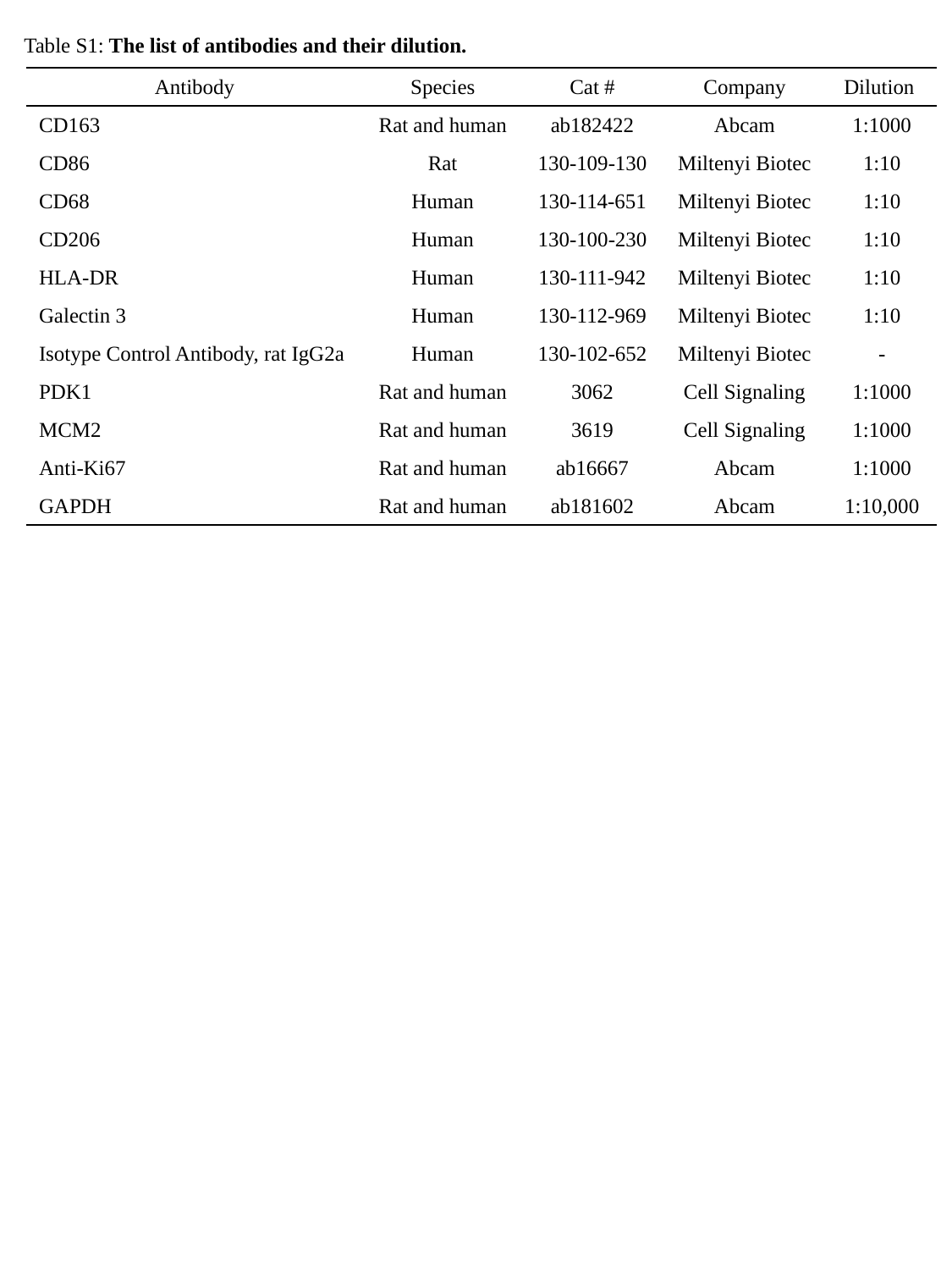

Table S1: The list of antibodies and their dilution.
| Antibody | Species | Cat # | Company | Dilution |
| --- | --- | --- | --- | --- |
| CD163 | Rat and human | ab182422 | Abcam | 1:1000 |
| CD86 | Rat | 130-109-130 | Miltenyi Biotec | 1:10 |
| CD68 | Human | 130-114-651 | Miltenyi Biotec | 1:10 |
| CD206 | Human | 130-100-230 | Miltenyi Biotec | 1:10 |
| HLA-DR | Human | 130-111-942 | Miltenyi Biotec | 1:10 |
| Galectin 3 | Human | 130-112-969 | Miltenyi Biotec | 1:10 |
| Isotype Control Antibody, rat IgG2a | Human | 130-102-652 | Miltenyi Biotec | - |
| PDK1 | Rat and human | 3062 | Cell Signaling | 1:1000 |
| MCM2 | Rat and human | 3619 | Cell Signaling | 1:1000 |
| Anti-Ki67 | Rat and human | ab16667 | Abcam | 1:1000 |
| GAPDH | Rat and human | ab181602 | Abcam | 1:10,000 |

## Slide 6
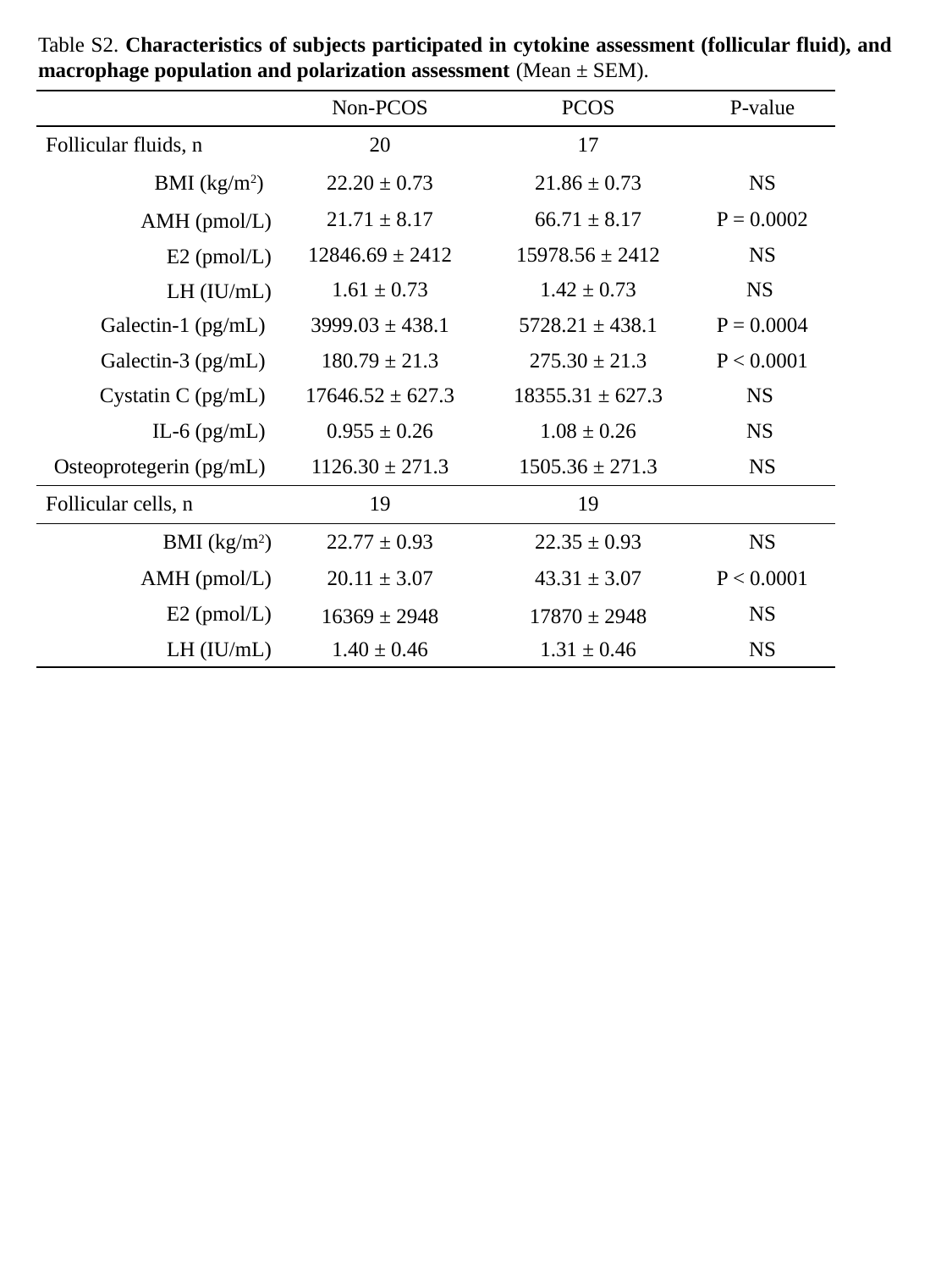

Table S2. Characteristics of subjects participated in cytokine assessment (follicular fluid), and macrophage population and polarization assessment (Mean ± SEM).
| | Non-PCOS | PCOS | P-value |
| --- | --- | --- | --- |
| Follicular fluids, n | 20 | 17 | |
| BMI (kg/m2) | 22.20 ± 0.73 | 21.86 ± 0.73 | NS |
| AMH (pmol/L) | 21.71 ± 8.17 | 66.71 ± 8.17 | P = 0.0002 |
| E2 (pmol/L) | 12846.69 ± 2412 | 15978.56 ± 2412 | NS |
| LH (IU/mL) | 1.61 ± 0.73 | 1.42 ± 0.73 | NS |
| Galectin-1 (pg/mL) | 3999.03 ± 438.1 | 5728.21 ± 438.1 | P = 0.0004 |
| Galectin-3 (pg/mL) | 180.79 ± 21.3 | 275.30 ± 21.3 | P < 0.0001 |
| Cystatin C (pg/mL) | 17646.52 ± 627.3 | 18355.31 ± 627.3 | NS |
| IL-6 (pg/mL) | 0.955 ± 0.26 | 1.08 ± 0.26 | NS |
| Osteoprotegerin (pg/mL) | 1126.30 ± 271.3 | 1505.36 ± 271.3 | NS |
| Follicular cells, n | 19 | 19 | |
| BMI (kg/m2) | 22.77 ± 0.93 | 22.35 ± 0.93 | NS |
| AMH (pmol/L) | 20.11 ± 3.07 | 43.31 ± 3.07 | P < 0.0001 |
| E2 (pmol/L) | 16369 ± 2948 | 17870 ± 2948 | NS |
| LH (IU/mL) | 1.40 ± 0.46 | 1.31 ± 0.46 | NS |

## Slide 7
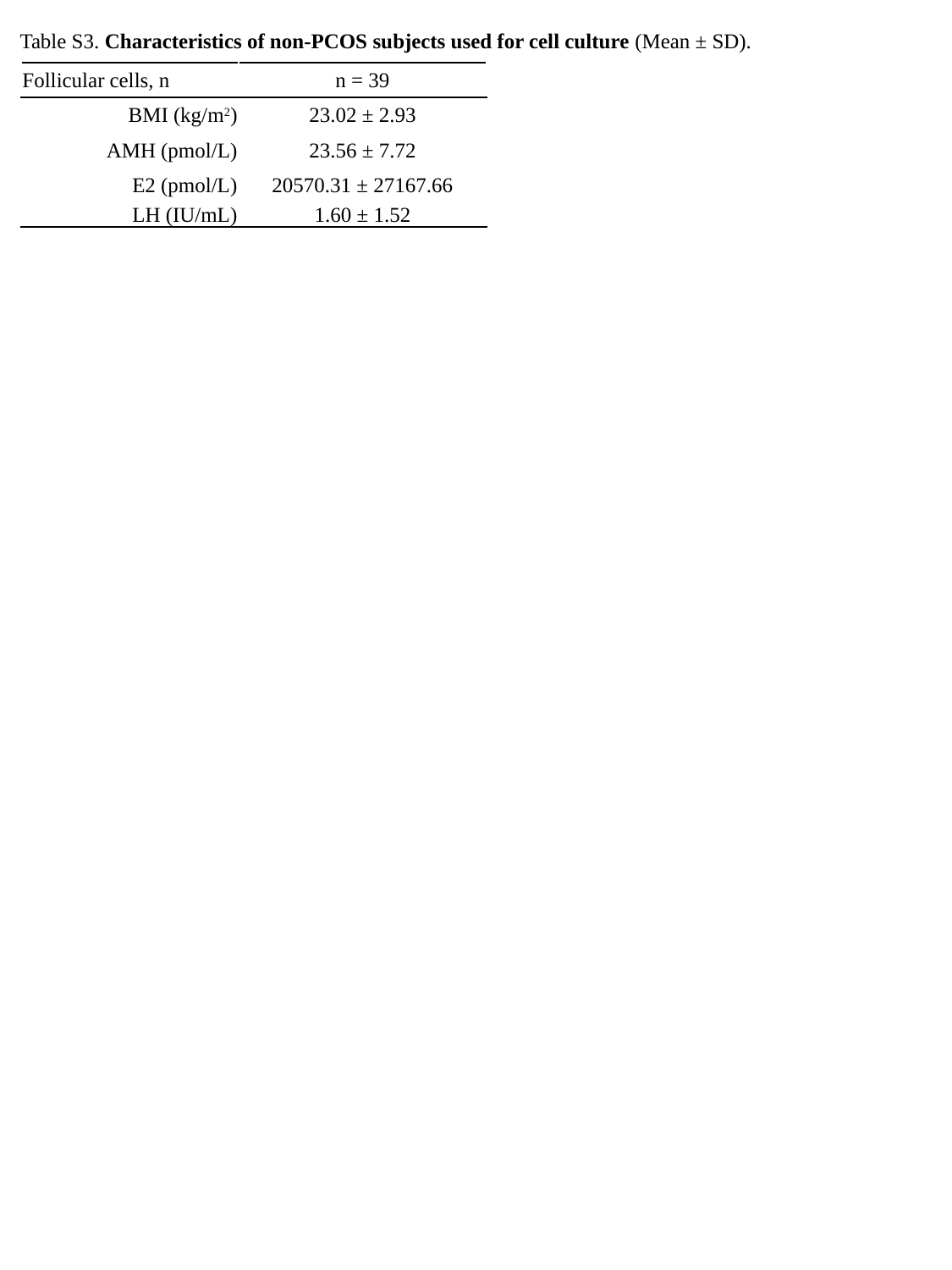

Table S3. Characteristics of non-PCOS subjects used for cell culture (Mean ± SD).
| Follicular cells, n | n = 39 |
| --- | --- |
| BMI (kg/m2) | 23.02 ± 2.93 |
| AMH (pmol/L) | 23.56 ± 7.72 |
| E2 (pmol/L) | 20570.31 ± 27167.66 |
| LH (IU/mL) | 1.60 ± 1.52 |
